# Cross-correlation beamforming

**DOI:** 10.1007/s10950-016-9612-6

**Published:** 2016-10-07

**Authors:** Elmer Ruigrok, Steven Gibbons, Kees Wapenaar

**Affiliations:** 1 Department of Earth Sciences, Utrecht University, Utrecht, The Netherlands; 2 R&D Seismology and Acoustics, Royal Netherlands Meteorological institute, De Bilt, The Netherlands; 3 NORSAR, Kjeller, Norway; 4 Department of Geoscience and Engineering, Delft University of Technology, Delft, The Netherlands

**Keywords:** Beamforming, Cross-correlation, Waveform characterization

## Abstract

An areal distribution of sensors can be used for estimating the direction of incoming waves through beamforming. Beamforming may be implemented as a phase-shifting and stacking of data recorded on the different sensors (i.e., conventional beamforming). Alternatively, beamforming can be applied to cross-correlations between the waveforms on the different sensors. We derive a kernel for beamforming cross-correlated data and call it cross-correlation beamforming (CCBF). We point out that CCBF has slightly better resolution and aliasing characteristics than conventional beamforming. When auto-correlations are added to CCBF, the array response functions are the same as for conventional beamforming. We show numerically that CCBF is more resilient to non-coherent noise. Furthermore, we illustrate that with CCBF individual receiver-pairs can be removed to improve mapping to the slowness domain. An additional flexibility of CCBF is that cross-correlations can be time-windowed prior to beamforming, e.g., to remove the directionality of a scattered wavefield. The observations on synthetic data are confirmed with field data from the SPITS array (Svalbard). Both when beamforming an earthquake arrival and when beamforming ambient noise, CCBF focuses more of the energy to a central beam. Overall, the main advantage of CCBF is noise suppression and its flexibility to remove station pairs that deteriorate the signal-related beampower.

**Electronic Supplementary Material:**

The online version of this article (doi:10.1007/s10950-016-9612-6) contains supplementary material, which is available to authorized users.

## Introduction

In various application areas, e.g., seismology, astronomy, and geodesy, arrays of sensors are used to characterize incoming wavefields due to distant sources. Beamforming is a general term for phase-adjusted summations over the different array elements. Beamforming may be used to steer the measured wavefield to a single direction (e.g., van Veen and Buckley 1988; Rost and Thomas 2002). By doing so, the beamformer acts as a spatial filter, yielding data with an improved signal-to-noise ratio for the phase-shifts specified. Alternatively, beamforming may be used to diagnose an array measurement. In this case, the phase-adjusted summations are performed for a range of backazimuths and slownesses; each backazimuth and slowness describes one plane-wave model. The models that show the greatest consistency with the measured wavefield lead to a large stacking power, which is called beampower in the following. Hence, a plot of the beampower is then used to unravel the directions of the measured wavefields. In this study, we use beamforming as a diagnostic tool.

Instead of applying phase shifts to the actual waveforms recorded on the different sensors, phase shifts can be applied to cross-correlations between these waveforms (Birtill and Whiteway 1965). In the latter case, wavefield models (usually plane-wave approximations) are sought which best explain the measured phase differences between the signals on the different array elements. An early advocate of this approach are Posmentier and Herrmann (1971), who demonstrated the statistical advantages of what they coined the “cophase” method. Their method, and small variations to it, became common in sound monitoring (e.g., Boone et al. 2000). In seismology, cross-correlations between waveforms on different sensors has long been employed to refine estimates of signal delay times (e.g., VanDecar and Crosson 1990). Frankel et al. (1991) employed a method akin to co-phase that they termed ZLCC (zero-lag cross-correlation) to scan the slowness parameter space. This method and related procedures have been applied to near-field (e.g., Almendros et al. 1999; Wassermann and Ohrnberger 2001; Cros et al. 2011; Hillers et al. 2014), regional (e.g., Gibbons et al. 2011), and global-scale (e.g., Landès et al. 2010) parameter estimation scenarios. A probabilistic expansion of ZLCC has been derived in Saccorotti and Del Pezzo (2000). Infrasound signals are typically coherent between neighboring sensors over far longer time-durations than seismic signals, which makes them particularly amenable to cross-correlation based processing. In the context of monitoring compliance with the Comprehensive Nuclear-Test-Ban Treaty (CTBT), infrasound signal detection at the prototype International Data Centre was originally based on a ZLCC-type estimator (Brown et al. 2002). However, this procedure has been replaced at the International Data Center by PMCC (progressive multichannel cross-correlation, Cansi 1995), which estimates parameters based upon the cross-correlation-derived time-delays with maximum power rather than on a more informative grid-search procedure.

In seismology, continuous data are routinely cross-correlated to find virtual responses as if there were a source at one of the receivers and the induced wavefield were detected by the other receivers. A detailed description of this method, called seismic interferometry, can, e.g., be found in Larose et al. (2006), Bensen et al. (2007), and Wapenaar et al. (2010). To assess the accuracy with which these responses are retrieved, it is necessary to estimate the spectral content of the input data. This can be done through beamforming (Stehly et al. 2006; Yao et al. 2009; Ruigrok et al. 2012; Draganov et al. 2013; Almagro Vidal et al. 2014). The availability of cross-correlated data makes it a straightforward to apply also the beamforming to these cross-correlated data. But does this yield the same results as beamforming the original data?

In this study, we compare beamforming cross-correlated data (cross-correlation beamforming or CCBF) with beamforming the original data (conventional beamforming or BF). We do not consider improvements to conventional beamforming, like Capon beamforming, since they can be applied both for BF and for CCBF. Nor do we discuss non-linear stacking methods in much detail, like *n*th root stacking (Muirhead 1968) and phase-weighted stacking (Schimmel and Paulssen 1997) since again they are equally applicable to both methods.

In the following, we start with reviewing a kernel for conventional beamforming. We continue by deriving a kernel for beamforming cross-correlated data and we assess how it relates to other beamforming methods which include cross-correlations. The mathematical descriptions form the basis for comparing the different methods. We compare resolution and aliasing by computing array-response functions. BF and CCBF are surprisingly similar, but for the fact that with BF, implicitly, also non-informative auto-correlations are included. After that, we study how the methods perform with different levels of non-coherent noise. Next, we illustrate the enhanced flexibility of CCBF by having control on individual receiver pairs. In the end, we compare BF and CCBF for estimating waveform characteristics from field data, and we study the effect of time-windowing cross-correlations prior to beamforming.

## Theory

In this section, we formulate an implementation of conventional beamforming in the frequency domain. Next, we introduce the expression for cross-correlation beamforming and show that it is similar to cross-correlation and stacking of phase-adjusted recordings. We continue by stating the expressions for the array-response functions (ARFs). In the last subsection, these ARFs are used to explain the link between the spatial sampling characteristics of an array and its resolving power in terms of resolution and aliasing.

### Conventional beamforming

We define **d**(**x** ^(*i*)^, *t*) = [*d*(**x** ^(1)^, *t*) *d*(**x** ^(2)^, *t*)...*d*(**x** ^(*n*)^, *t*)] as a two-dimensional data matrix with *ns* time samples and *n* receivers. Furthermore, ^(*i*)^ is a receiver index and **x** = [*x*_1_ *x*_2_ *x*_3_] denotes a position vector of a receiver. Hence, *d*(**x** ^(*i*)^,*t*) is the data vector recorded at location *i*. In the following, we assume the sensors to be placed on a flat surface, reducing the position vector to **x** = [*x*_1_ *x*_2_]. For further processing, we take the data to the temporal Fourier domain:

1 $$ d\left( \textbf{x}^{(i)},\omega\right)={\int}_{-\infty}^{\infty} d\left( \textbf{x}^{(i)},t\right) {e}^{-\iota \omega t} dt,  $$

where *ι* denotes the imaginary unit, *ω* = 2 *π* *f* is the angular frequency, and *f* is the frequency.

With conventional beamforming, the beampower *P* (as function of horizontal slowness *p*, backazimuth *𝜃*, and angular frequency *ω*_*o*_) is computed using the following equation:

2 $$ P(p,\theta,\omega_{o})=\left|\sum\limits_{i=1}^{n} d\left( \textbf{x}^{(i)},\omega_{o}\right) {e}^{\iota \textbf{x}^{(i)} \textbf{k}^{T} }\right|^{2},  $$

where the wave vector **k** is defined as

3 $$ \textbf{k}=[k_{E}\,k_{N}]=\omega_{o} p [\sin(\theta)\,\cos(\theta)],  $$

and *k*_*E*_ and *k*_*N*_ denote the wavenumber in the eastward and northward direction, respectively, and ^*T*^ denotes the transpose. Futhermore, *ω*_*o*_ is the frequency for which the beampower is computed. Either (2) is computed for only this frequency sample or the beampower is averaged over a small frequency bin around *ω*_*o*_ (see, e.g., Gal et al. 2014). Equation 3 states that a wavenumber vector is composed for each combination of slowness (here the reciprocal of horizontal apparent velocity) and backazimuth, which together define a plane wave. Equation 2 states that the delay times *τ* ^(*i*)^ = **x** ^(*i*)^ **k** ^*T*^ are computed for each wavenumber vector and each receiver. These delay times define—together with the frequency of interest—phase-shift terms that are applied to the data values *d*(**x** ^(*i*)^,*ω*_*o*_). Finally, beampowers are obtained by summing the phase-shifted data over all receivers and by taking the squared norm of this sum.

Note that the sum in (2) is an approximation of the two-dimensional spatial Fourier Transform. For a regularly sampled array, the distance between the array elements *dx* determines the Nyquist wavenumber, i.e., the maximum wavenumber that can be mapped unambiguously: *k*_Nyq_ = *π*/(2*d* *x*). For an irregularly sampled array of stations, the clear distinction between directly mapped wavenumbers and aliasing, vanishes.

### Cross-correlation beamforming

With cross-correlation beamforming, the data are first cross-correlated for all possible receiver pairs. In the frequency domain, the cross-correlations can be written as

4 $$ c\left( \textbf{x}^{(i)},\textbf{x}^{(j)},\omega\right) = d\left( \textbf{x}^{(i)},\omega\right) \left\{d(\textbf{x}^{(j)},\omega)\right\}^{*},  $$

where ^∗^ denotes the complex conjugate. The correlation function *c*(**x** ^(*i*)^,**x** ^(*j*)^,*ω*) is only evaluated for *i*≠*j*, i.e., all auto-correlations are left out, since they do not contain directivity information. Consequently, for *n* receivers, we have *q* = *n*(*n*−1) receiver pairs (of which *n*(*n*−1)/2 are unique) and *q* corresponding cross-correlations remaining. These are concatenated to a two-dimensional matrix (in case of multiple frequencies): **c**(*ω*) = [*c* ^(1)^(*ω*) *c* ^(2)^(*ω*) ... *c* ^(*q*)^(*ω*)], where, e.g., *c* ^(1)^(*ω*) = *d*(**x** ^(1)^,*ω*){*d*(**x** ^(2)^,*ω*)}^∗^ and *c* ^(*q*)^(*ω*) = *d*(**x** ^(*n*−1)^,*ω*){*d*(**x** ^(*n*)^,*ω*)}^∗^. With CCBF, the beampower $\check {P}$ is computed using the following equation:

5 $$ \check{P}(p,\theta,\omega_{o})=\left|\sum\limits_{k=1}^{q} c^{(k)}(\omega_{o}) {e}^{\iota \omega_{o} 2 {h}^{(k)} p\cos(\theta^{(k)}-\theta)}\right|,  $$

where *h* ^(*k*)^ are the half offsets (i.e., half of the receiver-pair separations), *𝜃* ^(*k*)^ are the receiver-pair azimuths, and ^(*k*)^ is a receiver-pair index. The derivation of the receiver-pair delay-time expression *τ* ^(*k*)^=2*h* ^(*k*)^(*p*cos(*𝜃* ^(*k*)^−*𝜃*)) can be found in the following subsection. The delay times are computed for each receiver-pair and for each plane-wave defined by *p* and *𝜃*. These delay times define—together with the frequency of interest—phase-shift terms that are applied to the cross-correlated data. Finally, beampowers are obtained by summing the phase-shifted cross-correlations over all receivers and by taking the absolute value (complex modulus) of this sum.

Note that in (5), no squared norm is taken, like in (2), since the cross-correlation of the data already yields an energy measure. To obtain a power quantity, a division would need to be made by the duration of the input data. In (2) and (5), we do not explicitly write this division.

When (5) is averaged over a frequency band with varying signal strength over this bandwidth, it is advantageous to replace the cross-correlation by a spectrally normalized cross-correlation, e.g., the cross-coherence:

6 $$ h\left( \textbf{x}^{(i)},\textbf{x}^{(j)},\omega\right) = \frac{d\left( \textbf{x}^{(i)},\omega\right) \left\{d(\textbf{x}^{(j)},\omega)\right\}^{*}}{|d\left( \textbf{x}^{(i)},\omega\right)| |d\left( \textbf{x}^{(j)},\omega\right)|}.  $$

CCBF (5) and the co-phase method (Posmentier and Herrmann 1971) are nearly identical. With co-phase, the exponential in (5) is replaced by a cosine. This cosine term is multiplied with a sum of the amplitude spectra at the different receivers. Equation 5, on the other hand, contains a multiplication of the amplitude spectra (which is hidden in the correlation function *c* ^(*k*)^(*ω*_*o*_)). The co-phase was written as a sum over a large frequency bandwidth, unlike a small frequency band, or a single frequency, like in (5). To assure equal contribution of the different frequencies, despite differences in amplitudes, with co-phase each monochromatic result is normalized with the sum of the amplitude spectra at the different receivers, instead of with the multiplication of the amplitude spectra, like in (6).

### Correlation beamforming

Frankel et al. (1991) suggested to first apply time shifts to the data and subsequently to cross-correlate the data for all possible receiver pairs. If we additionally take the absolute value, their approach amounts—in the frequency domain—to

7 $$\begin{array}{@{}rcl@{}} \hat{P}(p,\theta,\omega)&=&\left|\sum\limits_{i=1}^{n} \sum\limits_{j=1}^{n} d\left( \textbf{x}^{(i)},\omega\right) {e}^{\iota (\textbf{x}^{(i)} \textbf{k}^{T}} \right.\\ &&\left. \left\{d(\textbf{x}^{(j)},\omega) {e}^{\iota (\textbf{x}^{(j)}) \textbf{k}^{T} } \right\}^{*}{\vphantom{\sum\limits_{i=1}^{n}}}\right|. \end{array} $$

Equation 7 can be re-organized to

8 $$\begin{array}{@{}rcl@{}} \hat{P}(p,\theta,\omega)&=&\left|\sum\limits_{i=1}^{n} \sum\limits_{j=1}^{n} d\left( \textbf{x}^{(i)},\omega\right) \left\{d\left( \textbf{x}^{(j)},\omega\right)\right\}^{*} \right. \\&&\left. {e}^{\iota \left( \textbf{x}^{(i)} - \textbf{x}^{(j)}\right) \textbf{k}^{T}} {\vphantom{\sum\limits_{j=1}^{n}}}\right|. \end{array} $$

Further, by rewriting the vector (**x** ^(*i*)^ − **x** ^(*j*)^) to 2*h* ^(*ij*)^[ sin(*𝜃* ^(*ij*)^) cos(*𝜃* ^(*ij*)^)], where *𝜃* ^(*ij*)^ is the receiver-pair azimuth and *h* ^(*ij*)^ = |**x** ^(*i*)^−**x** ^(*j*)^|/2 is the half offset, we find

9 $$\begin{array}{@{}rcl@{}} \hat{P}(p,\theta,\omega) &=& \left|\sum\limits_{i=1}^{n} \sum\limits_{j=1}^{n} d\left( \textbf{x}^{(i)},\omega\right) \left\{d(\textbf{x}^{(j)},\omega)\right\}^{*} \right.\\&& \left. {e}^{\iota \omega 2{h}^{(ij)}p \left[ \sin\left( \theta^{(ij)}\right) \sin(\theta) + \cos\left( \theta^{(ij)}\right) \cos(\theta)\right]}{\vphantom{\sum\limits_{j=1}^{n}}} \right|. \\ \end{array} $$

Using the trigonometric product-to-sum identity:

10 $$ \sin(\theta^{(ij)}) \sin(\theta) + \cos(\theta^{(ij)}) \cos(\theta) = \cos(\theta^{(ij)}-\theta),  $$

leaving out the auto-correlations and computing the beampower only for a single frequency or frequency bin, we find again (5). Hence, applying phase shifts prior to cross-correlation is similar to applying phase shifts after cross-correlation.

Applying (7), (8), or (9) for beamforming in small frequency bands, we call correlation beamforming (CBF). The only difference between CBF and CCBF is that auto-correlations are left out from the latter.

Note that Frankel et al. (1991) do not apply cross-correlations for all possible time lags. Instead, they multiply the time-shifted seismograms in the time domain, which operation corresponds to zero-lag cross-correlation. Hence, the name of their method: zero-lag cross-correlation (ZLCC). All dominant contributions are at the zero time lag. Therefore, in practice, it makes little difference whether the cross-correlation is extended to larger time lags or not. This is shown in Section 6.

### Array response functions

To compare different beamforming approaches, we use the array-response functions (ARFs) (Birtill and Whiteway 1965). The ARF is the beampower for a given plane-wave model, array configuration and beamforming method. The ARFs can be computed prior to the actual installation of an array to assess the resolving power for the expected waveforms.

The directionality of a plane-wave wavefield is defined by **k**_*S*_ = *ω*_*o*_ *p*_*S*_[sin(*𝜃*_*S*_) cos(*𝜃*_*S*_)], where *p*_*S*_ and *𝜃*_*S*_ are the slowness and backazimuth of the wavefield. The backazimuth is defined with respect to the center of gravity of the array. For an array of receivers located at **x**, the phase delay of an amplitude-normalized monochromatic plane wave at receiver *i* reads as ${e}^{-\iota \textbf {x}^{(i)} (\textbf {k}_{S})^{T} }$. Using the conventional beamforming (BF) beampower expression (2), the ARF is obtained by substituting the data values with the plane wavefield expression, yielding:

11 $$ P(p,\theta,\omega_{o};p_{S},\theta_{S})=\left|\sum\limits_{i=1}^{n} {e}^{\iota \textbf{x}^{(i)} (\textbf{k}-\textbf{k}_{S})^{T} }\right|^{2}.  $$

For cross-correlated data, the (differential) amplitude-normalized monochromatic plane wavefield defined by *p*_*S*_ and *𝜃*_*S*_ can be expressed as ${e}^{-\iota \omega _{o} 2 {h}^{(k)} p_{S}\cos (\theta ^{(k)} - \theta _{S})}$. By substituting this cross-correlated data model in (5), the cross-correlation-beamforming (CCBF) ARF is obtained:

12 $$\begin{array}{@{}rcl@{}} &&{\kern-.7pc}\check{P}(p,\theta,\omega_{o};p_{S},\theta_{S})=\\ &&{\kern.7pc} \left|\sum\limits_{k\,=\,1}^{q} {e}^{\iota \omega_{o} 2 {h}^{(k)}(p\cos(\theta^{(k)}\,-\,\theta)\! -\! p_{S}\cos(\theta^{(k)}\,-\,\theta_{S}) )}\right|. \end{array} $$

The ARF for CBF (9) is the same as (12), with the exception that the summation incorporates the auto-correlations. Hence, instead of summing over *q* = *n*(*n*−1) receiver pairs, the summation is extended to *q* = *n* ^2^ combinations.

### Resolution and aliasing

In this section, we show an example of an ARF for cross-correlation beamforming. This ARF serves to illustrate the connection between the spatial element distribution and resolution and aliasing characteristics in the beampower domain.

**Fig. 1:**
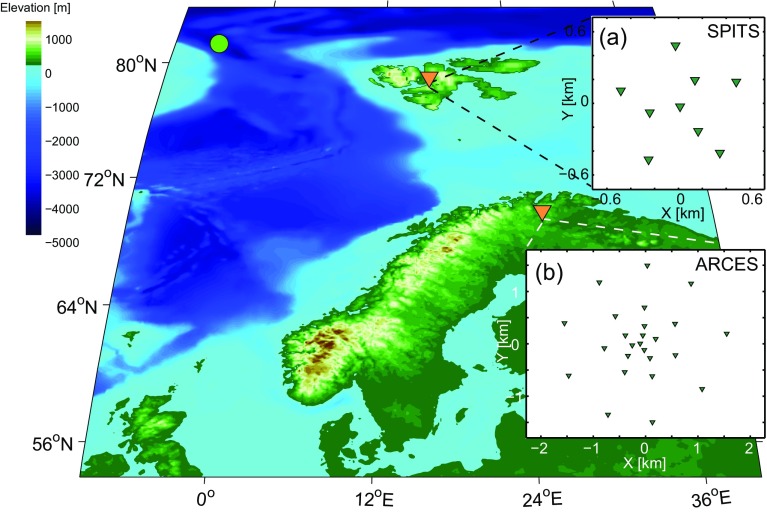
A regional overview of Svalbard and Scandinavia, including the locations and configurations of **a** the SPITS, **b** the ARCES array. The green circle denotes the location of the earthquake which response is beamformed in Section 6. *Orange triangles* denote the locations of the arrays, and *green triangles* denote seismic sensors within the arrays

**Fig. 2:**
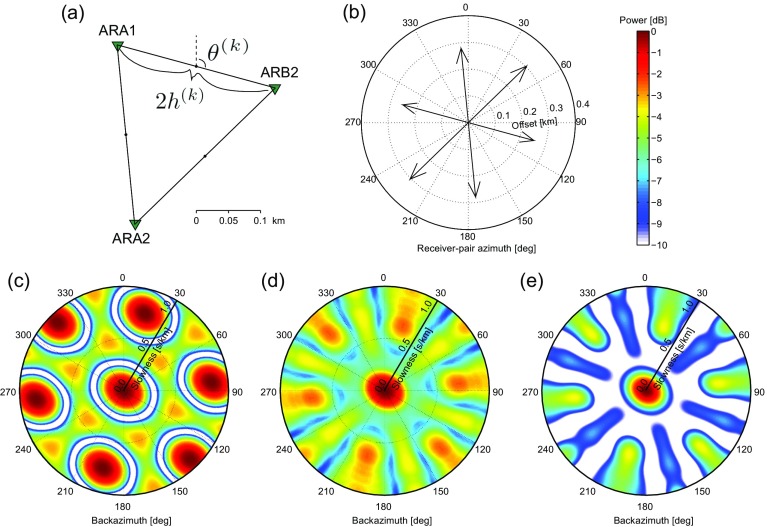
Array configuration, sampling and array-response functions(ARFs) for cross-correlation beamforming. **a** An array assembled by combining three stations (*green triangles*) of the ARCES array. The relevant parameters are indicated: 2*h* ^(*k*)^ denotes the offsets between different array elements and *𝜃* ^(*k*)^ denotes the azimuths of the lines connecting the different elements (receiver-pair azimuths). **b** A distribution plot showing the azimuthal and offset distribution of the receiver pairs in **a**. **c** ARF for beamforming a plane-wave source with *p*_*S*_=0 and *f*=5 Hz and **d** ARF for beamforming the same plane-wave source from 3 to 7 Hz. **e** As **d** but squared beampower instead of beampower is displayed. In **c**–**e,** the radial axes represent slowness, from 0 to 1.0 s/km

From the ARCES array (Mykkeltveit et al. 1990) in northern Norway (Fig. 1), we select stations ARA1, ARA2, and ARB2. Figure 2a shows the station distribution. The array samples a distribution of (receiver-pair) azimuths and offsets, which determine the resolution and aliasing in the *p*−*𝜃* domain for a given frequency. Each station pair samples one distance 2*h* ^(*k*)^ in two directions *𝜃* ^(*k*)^ & *𝜃* ^(*k*)^+180^∘^. Figure 2b displays the distribution of both parameters.

Figure 2c shows the CCBF ARF (12). As a source model, a plane wave is used with *f*=5 Hz, impinging the array from vertically below, that is with *p*_*S*_=0. The ARF is shown in a polar plot, where the slowness axis is along the radius and the backazimuth axis is along the circumference. In this and subsequent figures, the beampower is normalized using the maximum beampower within the plotted range. The ARF correcly maps a peak power at *p*=0. The power only slowly diminishes for larger slowness, which limits the resolution in the *p*−*𝜃* plane.

The resolution is dependent on the maximum receiver offset (i.e., the aperture of the array). This offset is azimuth dependent (Fig. 2b) and hence also the resolution is azimuth dependent. This can be seen in Fig. 2c: the area where the power remains high is an ellipse rather than a circle, with the minor semi-axis corresponding to the direction with the largest offset. For simplicity, we approximate the resolution slowness as a backazimuth independent function:

13 $$ p_{Res}=1/(4 h_{max} f),  $$

where 2*h*_*max*_ is the largest offset within the array. With *f*=5 Hz and 2*h*_*max*_=0.3 km (Fig. 2b), this yields *p*_*Res*_=0.33 s/km.

The aliasing is dependent on the minimum receiver offset, which also varies with azimuth. The aliasing is manifested in Fig. 2c by a repetition of beampower patterns: besides to the actual slowness, the plane wave is also erroneously mapped to higher slownesses. The Nyquist slowness, i.e., the slowness beyond which repetition occurs, we approximate with

14 $$ p_{Nyq}=1/(4 h_{min} f),  $$

where *h*_*min*_ is the smallest half-offset within the array. With *f* = 5 Hz and 2*h*_*min*_ = 0.25 km we find *p*_*Nyq*_ = 0.4 s/km. In Fig. 2c, aliasing maxima occur at ∼2*p*_*Nyq*_. As the aliasing, like the resolution, is in fact a direction dependent function, the aliasing artifacts appear precisely in between the azimuths that are sampled.

The Nyquist slowness is frequency dependent. Hence, if the signal is coherent over a band of frequencies, the mapping to the *p*−*𝜃* domain can be improved by stacking the beampower over this band. Figure 2d shows the resulting beampower for stacking the ARFs over a frequency range from 3 to 7 Hz (with increments of 0.5 Hz). Over this band, the signal is assumed to be stable in directivity. For higher frequency, *p*_*Nyq*_ goes down (14) and the aliasing thus moves towards the center of the *p*−*𝜃* domain. This frequency dependence of the aliasing leads to a smearing of the aliased beampowers. The maximum beampower related to the actual directivity of the signal is not frequency dependent. Consequently, stacking beampower over frequency leads to a power reduction of the aliasing artifacts and improves detectability of the actual directivity of the signal.

The *p*−*𝜃* domain plots in Fig. 2c, d, and plots alike, could visually be improved by not plotting the power, but the power squared, or even higher powers of the power. Since $10 \log _{10} \tilde {P}^{2}= 20 \log _{10} \tilde {P}$, the logarithm of the squared power difference between signal and artifacts becomes twice as big as that of the non-squared power difference. Figure 2e shows the power-squared version of 2e and the further reduction of artifacts can be appreciated here. Such non-linear improvement is worth considering when only the directivity of the dominant arrival is of interest. When the measurement contains wavefields with different directivity and amplitude, the non-linear enhancement will likewise amplify the primary beam with respect to the less energetic, but still physical, secondary beams. In the following, we will leave out such non-linear enhancements since they can equally be applied to enhance the output of BF, CBF, and CCBF.

## ARF comparison

**Fig. 3:**
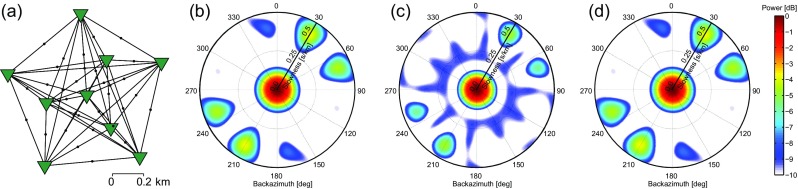
**a** The configuration of array elements (*green triangles*) of the SPITS array (Fig. 1) and **b**–**d** corresponding ARFs for varying beamforming implementations, for a plane-wave source with *p*_*S*_ = 0 and *f* = 5 Hz. ARF for **b** conventional beamforming, **c** cross-correlation beamforming, and **d** correlation beamforming. In **b**–**d**, the radial axes represent slowness, from 0 to 0.5 s/km

In this section, we compare the ARF for conventional, correlation, and cross-correlation beamforming. We use the station configuration of the SPITS array (Gibbons et al. 2011), which is a concentric distribution of nine stations. Figure 1 shows the location of the array. Figure 3a depicts the configuration of the array elements. The Supporting Material includes sampling characteristics of the SPITS array (Fig. SM1(b)).

The ARFs are computed using (11) and (12) for BF and CCBF, respectively. For CBF, we also use (12) as a base, but we expand the summation to include *n* auto-correlations. As a source model, a plane wave is used with *f* = 5 Hz, impinging the array from vertically below, that is with *p*_*S*_ = 0. Figure 3b–d shows the resulting ARFs.

Studying Fig. 3, it is obvious that the BF ARF (Fig. 3b) and the CBF ARF (Fig. 3d) are the same. The CCBF ARF (Fig. 3c) does have a slightly higher resolution than the two other methods. Note that the −9 dB ring (blue color) in Fig. 3c occurs for smaller slowness than in Fig. 3b, d.

Figure 3 shows that, from a sampling perspective, conventional beamforming (phase-shifting, summing and taking the squared norm; (2)) is identical to correlation beamforming (phase-shifting, correlating, and taking the norm; (7)). The higher resolution of CCBF with respect to BF and CBF must be ascribed to the omission of the auto-correlations.

The cause of the higher resolution can be found by studying the CCBF ARF expression (12). Including the auto-correlations to the summation would lead to the addition of *n* *e* ^*ιω*0^ = *n* to the beampower, for any slowness and backazimuth. This additional direction-independent raise of the beampower lowers the logarithmic difference between the power of signal and the power of spuriously mapped energy in the *p*−*𝜃* domain. As an effect aliasing goes up and resolution goes down.

Note that the cross-correlations do not result in an information gain. Neither is there redundancy in the cross-correlations if each station pair samples a different azimuth-offset combination. This is the case for the SPITS array (Fig. SM1(b)). Thus, removing a station pair from the SPITS array would deteriorate the ARF.

## Robustness

In this section, we assess the robustness of BF and CCBF in the presence of non-coherent noise. For this test, we use again the station configuration of the SPITS array (Svalbard).

We numerically model 164 s of data (16,384 samples) due to one continuously acting source located 40 km west of the array. This source itself acts as a noise source, but its wavefield is our signal of interest. Wave propagation in the horizontal plane is modeled, taking geometrical spreading into account. The modeling is done in a two-dimensional lossless homogeneous acoustic medium with a velocity of 3 km/s. The source has a peak frequency of 5 Hz. To the coherent (source-emitted) noise, random noise is added with varying signal-to-noise ratios (SNRs). We express the SNR in decibel:

15 $$ \text{SNR}=10 \log_{10} \frac{P_{S}}{P_{N}},  $$

where *P*_*S*_ is the power of the source-related wavefield and *P*_*N*_ is the power of the non-coherent noise. *P*_*S*_ and *P*_*N*_ are computed in the time domain as an average power over the entire duration of the synthetic recording (164 s). The noise is forward modeled in the same frequency band as the signal. The noise, however, has a nearly white distribution over *p*,*𝜃* space, whereas the signal is localized to (*p*_*S*_,*𝜃*_*S*_)=(0.33 s/km ,270^∘^).

**Fig. 4:**
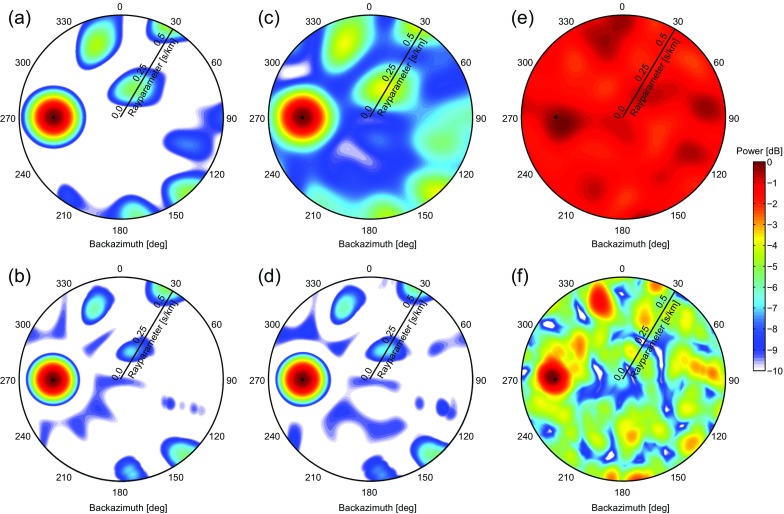
A comparison of BF (**a**, **c**, and **e** *upper row*) and CCBF (**b**, **d**, and **f** *lower row*) with increasing levels of non-coherent noise from left to right. Synthetic data are beamformed for a continuous noise source west of the SPITS array (Fig. 1). The parameters of the induced wavefield are marked with *black dots* at (*p*_*S*_,*𝜃*_*S*_)=(0.33 s/km ,270^∘^). The first column shows the beampower for BF (*up*) and CCBF (*down*) for an equal power of signal and noise (that is for an SNR (15) equal to 0 dB). The second and third column show the beampowers for SNR =−12 and −24 dB, respectively. The radial axes represent slowness, from 0 to 0.5 s/km

Figure 4(left) and (right) show the beampower as obtained with BF and CCBF, respectively, for the frequency band *f*=[46] Hz. For BF, the measurement is first chopped up in 36 segments and the beampower is averaged over these time segments. For CCBF, the cross-coherence (6) is applied instead of the cross-correlation, to ensure an equal contribution of the different frequencies. For BF, for the same reason, whitening is applied. The complete cross-correlations (from −164 to 164 s) are used for CCBF. The black dots in Fig. 4 show the actual parameters of the source with respect to the center of the array. The first, second, and third rows in Fig. 4 show the results for a SNR equal to 0, −12, and −24 dB, respectively.

For SNR =0 dB (Fig. 4a, b), both BF and CCBF yield a beampower distribution that is almost equal to the one that would be obtained without the presence of non-coherent noise (compare with Fig. 3). As before, CCBF has a slightly higher resolution in the *p*−*𝜃* domain. For SNR =−12 dB (Fig. 4c, d), the source parameters remain well estimated with both methods. For BF (Fig. 4c), the artifacts increase in power, while for CCBF (Fig. 4d) the artifacts remain nearly the same as for SNR =0 dB. For SNR =−24 dB (Fig. 4e, f), both BF and CCBF show a reduced gain. For BF (Fig. 4e), the noise mapped to the *p*−*𝜃* domain becomes of similar order as the signal, therefore limiting the detection capabilities, while for CCBF (Fig. 4f) the beampower related to the signal still stands out. However, interference with noise does somewhat deflect the maximum beampower from the location of the source.

CCBF turns out to be more robust than BF in the presence of non-coherent noise. With CCBF, the noise is largely suppressed in the cross-correlation process. The longer the input duration from the original data, the more the cross-correlation suppresses the non-coherent noise. Hence, similar noise reduction capabilities as in Fig. 4 would not be achieved when a small time-window around a transient signal is selected for beamforming. With BF, the non-coherent noise is suppressed by averaging over multiple time segments. The source beam is coherent from segment to segment, whereas the directionality of the non-coherent noise varies from segment to segment. Also for BF, the noise-reduction capabilities get worse for a shorter duration of the input signal because averaging would take place over less segments.

For the above examples (Fig. 4), the computation time of BF and CCBF is 3 and 11 s, respectively, on an off-the-shelf 2015 laptop. For CCBF, this includes the computation time for the cross-correlations. When an array has *n* stations, for CCBF the computation time scales with *n*(*n*−1)/2, while for BF it only scales with *n*. For arrays with many more than nine elements, BF would thus be preferred in view of the shorter computation time. However, for CCBF, the computation time could be reduced by removing near-redundant station pairs. In the above examples, BF requires more computation time than CCBF, mainly due to averaging the beampower over 36 time segments for BF. A larger amount of samples than 16,384 would further slow down BF. Hence, for studying the directivity of a noise field over hours or days, CCBF would be faster to compute than BF, especially when cross-correlations are already available for a different purpose.

## Flexibility

In this section, we illustrate the enhanced flexibility of CCBF with respect to BF using a T-shaped array.

**Fig. 5:**
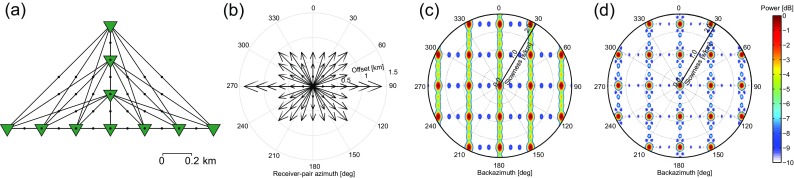
Sampling and beamforming characteristics for an upside-down T-array. **a** Spatial configuration of the array with ten receivers (*green triangles*), with interconnecting lines between all receivers depicted. **b** Distribution plot showing the azimuth and offset distribution of the receiver pairs in **a**. **c** BF ARF and **d** CCBF ARF for beamforming a plane-wave source with *p*_*S*_=0 and *f*=5 Hz. In **c** and **d**, the radial axes represent slowness, from 0 to 2.0 s/km

T-arrays are logistically convenient, especially for temporary arrays. They can be deployed from two intersecting roads (Fig. 5a) and still result in a fairly regular distribution in receiver-pair azimuths and offsets (Fig. 5b). In seismology, a T-shaped design has, e.g., been used for the Yellowknife array (Rost and Thomas 2002).

Figure 5c, d shows ARFs for BF and CCBF, respectively. Due to the regular sampling, *p*_*Nyq*_ (14) is well defined and the beampower is precisely repeated beyond *p*_*Nyq*_, rather than creating aliasing artifacts with a reduced power, as for non-regularly sampled arrays. Hence, for the T-array, the aperture in the *p*−*𝜃* domain is limited. This might be a reason that T-shaped arrays were frequently used in the past (Birtill and Whiteway 1965), but have largely been replaced by concentric arrays (Rost and Thomas 2002). For a discussion of different array designs and their pros and cons, see the Supporting Material.

Besides the repetitions, the T-array BF ARF (Fig. 5c) shows linear aliasing artifacts. These artifacts find their origin in oversampling. The 0^∘^,90^∘^,180^∘^, and 270^∘^ azimuths are over represented (Fig. 5b). For example, the smallest offset is sampled six times in the 90^∘^ and 270^∘^ directions and three times in the 0^∘^ and 180^∘^ directions.

As we have found in Section 3, BF and CCBF have similar ARF. However, the resolution and level of aliasing is somewhat better for CCBF due to the omission of autocorrelations. Thus, also for the T-array CCBF ARF, the same repetitions and linear aliasing features exist as in Fig. 5c. However, for CCBF, we have the flexibility to improve the array response by removing the oversampling. In Fig. 5d, we show the CCBF array response after removing all the non-unique offsets at the aforementioned azimuths. This improved ARF does not contain the linear features that appear on Fig. 5c.

For BF, we have the flexibility to remove array elements from the data analysis, if this is beneficial for the data analysis. For example, data can be left out from elements with poor SNR. For CCBF, we also have the flexibility to remove array elements from the data analysis. Besides, we have the flexibility to remove individual receiver pairs, e.g., to improve the ARF from an array that has been installed already. In this section, we have shown that the latter may allow mitigation of aliasing while maintaining the same resolution.

## Field data

In this section, we study the behavior of CCBF in comparison with BF for a field data case. Another field-data comparison can be found in Gibbons et al. (2015). We use the actual vertical particle-velocity recordings from the SPITS array (Fig. 1a). Because one station had timing issues (the one at the southwestern edge of the array), we use the recordings of the eight remaining stations. As with the synthetic data in the previous section, we beamform the data over a frequency range of *f*=[46] Hz, using five frequency bins. As a benchmark, we first beamform data from a known earthquake. Secondly, we beamform ambient noise.

**Fig. 6:**
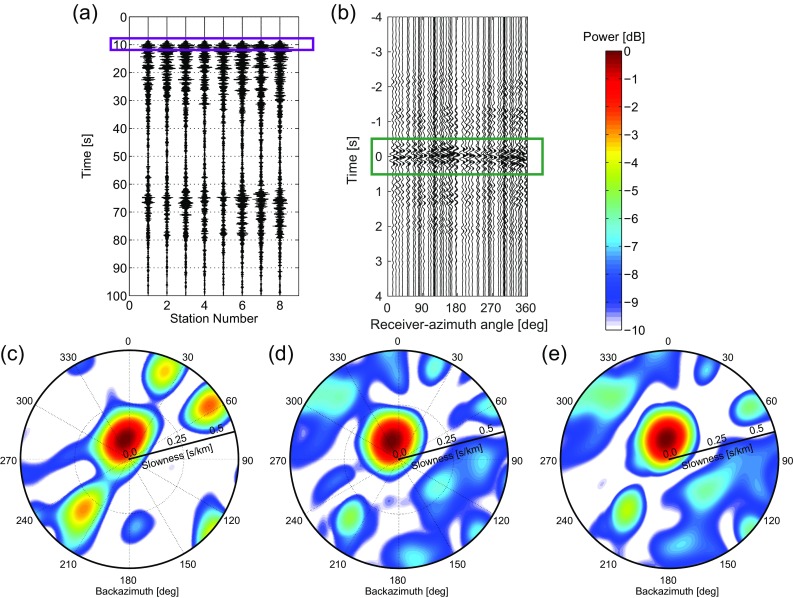
An earthquake beamform example with data from the SPITS array (Fig. 1). **a** A regional earthquake response over the array. The *purple box*indicates a 4 s time-window around the first P-wave arrival. **b** is the result of cross-correlating the data in **a** within the *purple box*, for all receiver pairs. The *green box* indicates a time-window from −0.5 to 0.5 s. Beamforming the data in the purple box in **a** results in **c** the BF power. Beamforming the cross-correlated data in **b** results in **d** the CCBF power distribution. Taking instead of the complete cross-correlations only the portion within the *green box* in **b** results in **e** the time-windowed CCBF power distribution. In **c**–**e**, the radial axes represent slowness, from 0 to 0.5 s/km

The earthquake (mb 4.4, 2013-02-01 11:31:19.0 UTC) is located by the European-Mediterranean Seismological Centre in the ocean-spreading ridge between Svalbard and Greenland (Fig. 1). The location is at 561 km from the center of the SPITS array and at a backazimuth of 324^∘^. We select a 4 s time-window around the first P-wave arrival (Fig. 6a) as input for the beamforming algorithms. Figure 6c, d shows the resulting beampowers for BF and CCBF, respectively. With BF, the waveform is picked as (*p*_*S*_, *𝜃*_*S*_) = (0.095 s/km, 348^∘^), while CCBF has a maximum beampower at (*p*_*S*_, *𝜃*_*S*_) = (0.090 s/km, 339^∘^).

In Fig. 6b, the intermediate step for CCBF is shown, that is the cross-correlated data for all receiver pairs. The main feature in this panel, around *t* = 0, exhibits by approximation a cosine behavior (5). The receiver-pair azimuth at which the maximum negative time lag occurs corresponds to the backazimuth of the source. The feature around *t* = 0 largely originates from the cross-correlation of direct waves. Cross-correlation of direct waves and scattered waves would end up at larger time lags. Also if the wavefield is coherent in time, that is, if the wavefield repeats after a fixed duration, this leads to additional features at larger time lags. Both contributions from scattering and wavefield repetitions can be largely excluded by taking, instead of the complete cross-correlation result, only this main contribution around *t* = 0 (as indicated by the green box in Fig. 6b). This yields the time-windowed CCBF result as depicted in Fig. 6e. This could be seen as an approximation of the ZLCC (Section 1). Other examples of time-windowing cross-correlation functions to separate different contributions can be found in Wapenaar et al. (2011).

Both BF and CCBF do a reasonable job in finding the backazimuth of the source. However, the slowness estimates are quite far off from the theoretically expected value of 0.12 *s*/*k* *m* based on a 1D reference model. It is known that wavefronts arriving at SPITS from the NNW reach larger apparent velocities than may be expected based on a 1D velocity model (Fig. 6 in Gibbons et al. (2011)). This is likely due to dipping interfaces in the crust below SPITS. Moreover, to further improve the directivity estimate, for both methods also corrections would need to be made for elevation differences over the SPITS array (Gibbons et al. 2011).

The CCBF beampower shows less severe artifacts. This is probably due to less sensitivity to non-coherent noise over the array (Section 4). For example, scattering near the array does not show a plane-wave coherency over the array and would be seen by the beamformer as non-coherent noise. Figure 6d, e is almost identical. Thus, for this data, time-windowing the cross-correlations does not improve the resolving power in the *p*−*𝜃* domain. This can be understood by the cross-correlations having most of their energy around *t*=0 and later coherent contributions having similar directivity as this feature around *t*=0 (Fig. 6b).

**Fig. 7:**
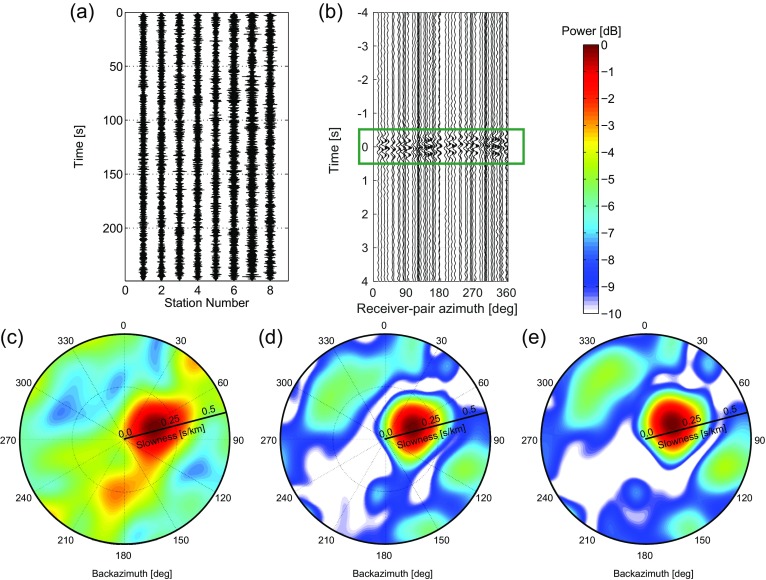
An earthquake beamform example with data from the SPITS array (Fig. 1). **a** A local noise response over the array. **b** is the result of cross-correlating the data in for all receiver pairs. The *green box*indicates a time-window from −0.5 to 0.5 s. Beamforming the data in **a** results in **c** the BF power. Beamforming the cross-correlated data in **b** results in **d** the CCBF power distribution. Taking instead of the complete cross-correlation result only the portion within the *green box* in **b** results in **e** the time-windowed CCBF power distribution. In **c**–**e**, the radial axes represent slowness, from 0 to 0.5 s/km

Next, we beamform a time interval of 250 s (starting at 1-1-2013, 1:45:00 UTC). In this time interval and for the frequency band of consideration (*f*=[46] Hz), only ambient noise is recorded, without any transient event that is distinguishable in the time domain (Fig. 7a). Figure 7c, d shows the resulting beampower plots for BF and CCBF, respectively.

Also the intermediate step for CCBF is shown, that is the cross-correlated data: Fig. 7b. Instead of showing the complete cross-correlation result (from −250 to 250 s) only a time-window is shown with the main contributions. As in Fig. 6b, a cosine-like feature can be distinguished around *t*=0. The azimuthal shift of this cosine feature is related to the backazimuth of the dominating source; the amplitude (maximum time shift in this case) is related to the appararent velocity of the dominant wave (5). Using only this main contribution around *t*=0 for CCBF results in the beampower as shown in Fig. 7e.

The CCBF beampower shows well-focused energy with a maximum at *𝜃*=66^∘^ and *p*=0.14 s/km (7.14 km/s). The BF beampower is less focused and has a maximum at *𝜃*=68^∘^ and *p*=0.155 s/km (6.45 km/s). Moreover, there is considerable energy all over the evaluated part of the *p*−*𝜃* domain. This may due to sources closer to the array (with circular wavefronts over the array). As with the earthquake P-wave arrivals, using only the main feature of the cross-correlation gives an almost identical result to using the complete cross-correlation result.

The origin of the noise source is unknown to us. It might be related to mining activity on Svalbard. Considering that the central beam for CCBF has a higher SNR than for BF, likely the directivity estimate for CCBF is more correct.

## Conclusions

In the foregoing, we compared straighforward beamforming of the recorded data with beamforming of the cross-correlated data. The former method we referred to as conventional beamforming (BF) and the latter as cross-correlation beamforming (CCBF).

We found that the array-response functions (ARFs) for BF and CCBF are different. With BF autocorrelations are, implicitly, included, which do not contain any information about the directivity of the source. The auto-correlations do reduce the gain. Consequently, for BF the resolution is a bit less and the aliasing artifacts are a bit stronger than for CCBF.

Using synthetic data, we showed that CCBF performed markedly better than BF when non-coherent noise was measured in addition to coherent wavefields. The non-coherent noise is drastically suppressed in the cross-correlation process.

Another advantage of CCBF was shown to be its higher flexibility in comparison with BF. For BF, one could decide to leave out data from individual elements. For CCBF, one could additionally leave out data from specific station pairs. Not only the auto-correlations can be left out, as is the norm for CCBF, but any receiver-pair that deteriorates the signal-related beampower. For example, when there are tiny timing issues between the instruments, it could be favorable to leave out the station pairs with the highest sensitivity to the timing errors (the ones at close range). On the other hand, when phases are not recorded coherently over the entire array, receiver-pairs at large range could be removed. For a T-shaped array, we showed that it is advantageous to remove station pairs that non-uniquely sample offset-azimuth combinations.

The observations on synthetic data were confirmed with field data from the SPITS array. Both when beamforming an earthquake arrival and when beamforming ambient noise, CCBF focused more of the energy to a central beam. We showed that cross-correlated data may be time-windowed prior to beamforming, which constitutes another flexibility of CCBF. Time-windowing would be advantageous to exclude scattered arrivals from the direct noise field. For the field-data cases, however, the beamforming results did not change notably after time-windowing, indicating that near-array scattering is no issue for the SPITS array for the frequency band considered.

## Electronic supplementary material

Below is the link to the electronic supplementary material.

(PDF 1.88 MB)
