# Supplementary material for: Cross-correlation beamforming
Source: J Seismol. 2016 Oct 7;21(3):495–508. doi: 10.1007/s10950-016-9612-6 (PMC5443858; doi:10.1007/s10950-016-9612-6)
Supplement: Supplementary file 1 — (PDF 1.88 MB) [file 10950_2016_9612_MOESM1_ESM.pdf]

Supporting Material for "Cross-correlation beamforming": array design

Elmer Ruigrok, Steven Gibbons and Kees Wapenaar

September 16, 2016

The SPITS and ARCES arrays are specifically optimized for conventional beamforming (Mykkeltveit et al, 1990). The SPITS array may be considered as the core of a log-spiral array. LOFAR (van Haarlem et al, 2013) is a recently built radio-astronomy array that fully expresses the log-spiral configuration. Going from the centre to the outer element along one spiral, each element is shifted in (array-centre to element) azimuth. Doing so, oversampling of specific azimuths is circumvented.

There may be many circumstances in which such optimal array designs are not feasible to implement in practice due to logistical considerations. In the following we consider three arrays that are logistically simple and assess their beamforming potential, both for conventional beamforming (BF) and cross-correlation beamforming (CCBF). For the definitions of BF and CCBF and their array response functions (ARFs), we refer to the main text.

Fig. 1(a) shows the spatial distribution of the sensors, with —in addition— the interconnecting lines. The distribution of the azimuths and offsets sampled by these lines (Fig. 1b) determine the BF ARF (Fig. 1c) and CCBF ARF (Fig. 1d). The ARFs are shown for a planar wavefield with a slowness equal to 0 s/km and with a slowness axis extending to 2.0 s/km. The second to fourth column in Fig. 1 show alternative arrays designs, their effective sampling and ARFs. The other 3 arrays are designed such that the maximum receiver-pair offset $2h$ is similar to the one in the SPITS array, which is ~ 1 km. This ensures that for each array a similar resolution is achieved (equation 13 in the main text). Setting an equal resolution by design, the quality of the array can be judged solely on the level of aliasing. The first 3 arrays consist of 9 elements, the 4th array has 10 elements.

The second array: the U-array (Fig. 1e) is a regular distribution of the 9 elements over a half circle with a diameter of 1 km. This design leads to a wide and quite regular distribution of azimuths and offsets being sampled (Fig. 1f) and consequently little aliasing in the ARFs (Fig. 1g&h). A reduction in offset sampling towards 0° and 180°, however, leads to an asymmetry in both the resolution and the aliasing. This asymmetry could be fixed by distributing the array elements over a half ellipse defined by $X = Rcos(\phi)$ and $Y = -2Rsin(\phi)$, where $R = 1$ km and ϕ is a regular distribution from 0 to π. For the latter design, though, an even sampling in ϕ leads to an uneven sampling in offset and hence to more aliasing in the ARFs.

With the third array: the C-array (Fig. 1i) the array elements are regularly distributed over a circle with a diameter of 1 km. This design leads to a very regular distribution in sampled receiver-pair azimuths and somewhat less regular distribution in offsets (Fig. 1j). Consequently, the ARFs (Fig. 1k&l) are point symmetric, but some serious aliasing (about -4 dB with respect to the peak power which is at 0 dB) already appears near $p = 0.65$ s/km. These aliasing artifacts are precisely in between the azimuths being sampled.

The fourth array is a variant on the T-array (see main text): the Y-array. The array we consider is in fact an upside-down Y with an 120° angle between the different arms (Fig. 1m). This design leads to a hexagram-shaped offset-azimuth distribution (Fig.

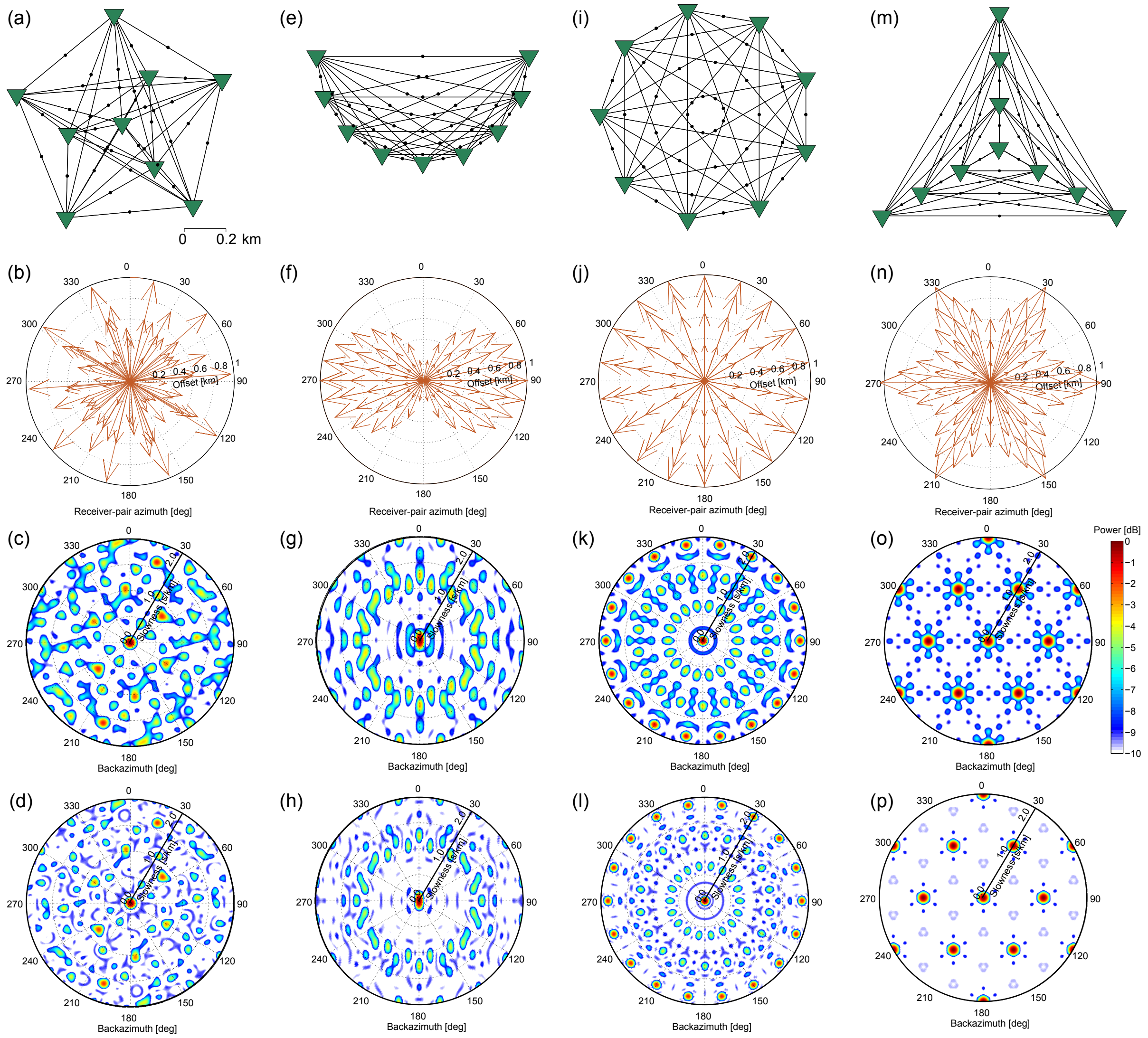

Figure 1: Comparing array response functions (ARFs) for $f = 5Hz$ and a planar wavefield with a slowness of 0 s/km for different array designs. The first row shows the array designs, with array elements (green triangles), the interconnecting lines (black lines) and midpoints (black dots): (a) the SPITS array (Gibbons et al, 2011), (e) the U-array, (i) the C-array and (m) the Y-array. The second row depicts polar distributions of the sampling (in receiver-pair azimuth and offset) achieved with the different array designs. The third and fourth row show the BF and CCBF ARFs, respectively, for the different array designs. On these panels, the radial axes represent slowness, from 0 to 2.0 s/km.

1n). In radio astronomy Y-shaped arrays were common, like the Very Large Array in New Mexico, USA (Thompson et al, 1980). Y-arrays, like T-arrays have aliasing artifacts as an exact repetition of the primary beam beyond the Nyquist slowness. However, Y-shaped arrays do not result in as much repeated sampling of the same receiver-pair azimuths & offsets as the T-array (Y has three primary directions, while T has two) and thus yield an improved ARF with respect to the T-array (Fig. 1o&p, compare with Fig. 5 in the main text).

Comparing the ARFs for BF and CCBF (row 3 and 4 in Fig. 1, respectively), both beamforming approaches yield similar beampower distributions. For CCBF, the resolution is slightly higher and the aliasing artifacts show a slightly reduced power with respect to ones for BF, which can be explained by the omission of auto-correlations for CCBF (Section 3 in main text).

Comparing the different array designs, the U-array is probably the most favorable for waveform characterization. This array achieves the largest ratio between the largest offset in the array $2h_{max}$ and the smallest offset in the array $2h_{min}$. This design thus yields the largest p_{Nyq} (equation 14 in main text), for a fixed p_{Res}. Indeed, Fig. 1(h) shows the least aliasing artifacts. For a slowness range until $p = 1s/km$, the U-array shows aliasing up till $-7dB$, whereas the SPITS array ($-3dB$), the Y-array ($0dB$) and the circular array ($-4dB$) show more serious aliasing. This aliasing is specifically relevant when multiple wavefields are to be characterized within the same data.

A disadvantage of the U-array is that the resolution is azimuth dependent. With the open end of the U pointing north (like in Fig. 1e), the highest resolution is obtained in the east-west directions. So, the U needs to be employed such that it points away from the direction from which most arrivals are expected.

A nice feature of the circular array is the point-symmetry of the aliasing. This quality makes it relatively easy to make corrections for the ARF and to take into account ARF perturbations due to a non-homogeneous subsurface below the array.

References

Gibbons SJ, Schweitzer J, Ringdal F, Kværna T, Mykkeltveit S, Paulsen B (2011) Improvements to Seismic Monitoring of the European Arctic Using Three-Component Array Processing at SPITS. Bulletin of the Seismological Society of America 101(6):2737–2754, DOI 10.1785/0120110109, URL http://dx.doi.org/10.1785/0120110109

van Haarlem MP, Wise MW, Gunst AW, Heald G, McKean JP, Hessels JWT, de Bruyn AG, Nijboer R, Swinbank J, Fallows R, et al (2013) LOFAR: The LOw-Frequency ARray URL http://arxiv.org/abs/1305.3550, preprint posted on http://arxiv.org/abs/1305.3550, 1305.3550

Mykkeltveit S, Ringdal F, Kværna T, Alewine RW (1990) Application of regional arrays in seismic verification research. Bulletin of the Seismological Society of America 80(6B):1777–1800

Thompson AR, Clark BG, Wade CM, Napier PJ (1980) The Very Large Array. The Astrophysical Journal Supplement Series 44:151–167, DOI 10.1086/190688, URL http://dx.doi.org/10.1086/190688
